# Three step synthesis of single diastereoisomers of the vicinal trifluoro motif

**DOI:** 10.3762/bjoc.5.61

**Published:** 2009-11-05

**Authors:** Vincent A Brunet, Alexandra M Z Slawin, David O'Hagan

**Affiliations:** 1 School of Chemistry and Centre for Biomolecular Sciences, University of St Andrews, North Haugh, St Andrews, Fife, KY16 9ST, UK

**Keywords:** C–F bond, organo–fluorine chemistry, stereospecific fluorination, vicinal trifluoro motif

## Abstract

A three step route to single diastereoisomers of the vicinal trifluoromethyl motif is described. The route starts from either *syn*- or *anti*-α,β-epoxy alcohols and takes a direct approach in that each of the three steps introduces a fluorine atom in a regio- and stereo-specific manner. Starting from either the *syn*- or the *anti*-α,β-epoxy alcohol, stereospecific reactions generate two separate diastereoisomeric series of this motif. The route is a significant improvement on an earlier six step strategy.

## Introduction

**Scheme 1:**
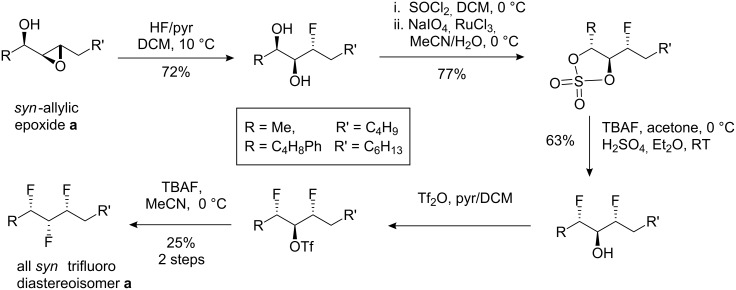
Previous six step route to the vicinal all-*syn*-trifluoro motif.

Selective fluorination is an important strategy for the design of performance molecules in medicinal chemistry programmes and in organic materials [1–2]. To date arylfluorines have dominated this agenda. However molecules where the C–F bond is a constituent of a stereogenic centre are gaining in prominence, particularly as new reagents and more versatile asymmetric methods facilitate their syntheses [3–4]. The fluorine atom is small, with a steric impact only a little larger than hydrogen, and it is a weak hydrogen bond acceptor [4]. However the C–F bond is polar and thus interactions with nearby functional groups are largely a result of dipolar interactions rather than hydrogen bonding or sterics. We have focused a recent synthetic effort on the assembly of multivicinal fluorine motifs where contiguous fluorines have been placed along alkane chains [5]. It emerges that different diastereoisomers of otherwise constitutionally identical isomers have very different properties and conformations as a consequence of the preferred alignments of the C–F bonds, and thus the specific stereogenic relationship between the vicinal fluorines alters the properties of the compounds in a very specific manner [6–10]. Earlier contributions in this area outlined a synthetic approach to the vicinal trifluoro motif as shown in Scheme 1 [11–12]. However this method had some limitations and particularly the final step was susceptible to competing elimination, resulting in poor yields. The route also required six steps to insert the three fluorine atoms, with a poor overall yield. A more practical route to this class of compounds is required if these vicinal trifluoro motifs are to be applied usefully.

**Scheme 2:**
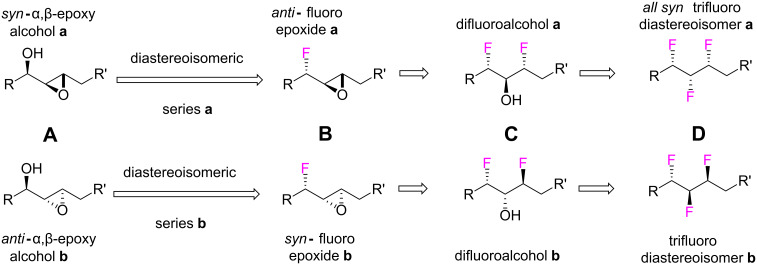
Novel three step successive fluorination strategy from α,β-epoxy alcohols to different diastereoisomeric series (**a** and **b**) of the vicinal trifluoro motif.

A three step strategy, as illustrated in Scheme 2, starting from diastereoisomeric *syn*- or *anti*-α,β-epoxy alcohols **A** was envisaged, each step incorporating a fluorine atom in a stereospecific manner. Conversion of the free hydroxyl group to fluorine would generate α-fluoro-epoxides **B**. Epoxide ring opening with an HF source could then provide difluoroalcohols **C**. Insertion of the third fluorine would reasonably be achieved by fluorination of the free hydroxyl group of **C** to generate **D**. Such a strategy offers a three step route to the vicinal trifluoroalkane motif and would avoid the use of TBAF, reducing the risk of elimination reactions competing with fluorine substitution, a problematic aspect of the last step in the earlier route shown in Scheme 1.

It was attractive to incorporate a peripheral tosyl group into the developing trifluoro moiety. The tosyl group was recently shown to be compatible with Deoxo-Fluor^®^ mediated deshydroxyfluorination reactions [7] and this would allow the prepared trifluoroalkyl motifs to be appended to more complex structural architectures in due course.

## Results and Discussion

**Scheme 3:**
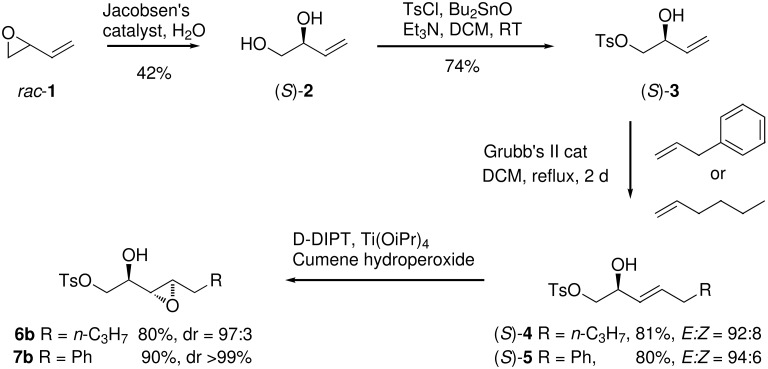
Synthesis approach to the requisite α,β-epoxy alcohols **6b** and **7b**.

The chemistry was initiated from allylic alcohol **2** which can readily be prepared in enantiomerically pure form by hydrolytic kinetic resolution of vinyl epoxide **1** following Jacobsen’s protocol [13] (Scheme 3).

**Figure 1:**
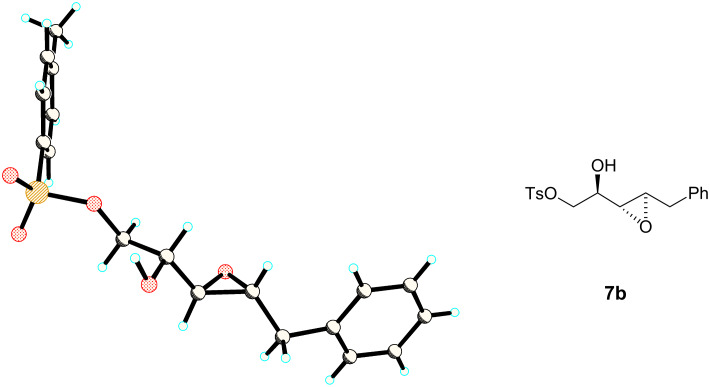
X-ray structure (CCDC 750307) and stereochemistry of α,β-epoxy alcohol **7b**.

Diol **2** was converted to tosyl ester **3** in a regioselective manner following the procedure of Marinelli [14]. The tosyl ester **3** was then submitted to two cross-metathesis reactions using the Grubbs II catalyst and with an excess of either hexene or allyl benzene (5 equiv) to form allylic alcohols (*S*)-**4** and (*S*)-**5** respectively [15]. The excess of alkene favours the cross metathesis reaction and the products were obtained in good yields predominantly as the (*E*)-isomer. It was not possible to separate the minor (*Z*)-isomer at this stage, however isomer separation was more readily achieved after the subsequent epoxidation step. Thus (*S*)-**4** and (*S*)-**5** were subjected to epoxidation reactions following Sharpless’ methodology [16]. The *anti*-epoxy alcohols were easily generated, however it proved more difficult to prepare the *syn*-epoxides cleanly. Treatment with titanium isopropoxide (Ti(OiPr)_4_) and D-diisopropyl tartrate (D-DIPT) favoured formation of the *anti*-α,β-epoxy alcohols. In the case of **4**, epoxide **6b** was obtained in 80% yield and in 97:3 dr. For **5**, the resulting epoxide **7b** was generated as the only observable diastereoisomer. Epoxide (2*R*,3*R*,4*R*)-**7b** was recrystallised from diethyl ether to afford a suitable crystal for X-ray structure analysis which confirmed its relative and absolute configuration (Figure 1).

**Figure 2:**
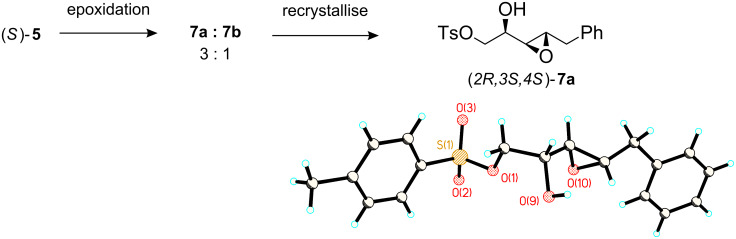
X-ray structure (CCDC 750306) and stereochemistry of α,β-epoxy alcohol **7a**.

Generation of the *syn*-α,β-epoxy alcohols **Aa** was more challenging. This stereoisomer will ultimately deliver the *all-syn* **Da** trifluoro motif (Scheme 1). Epoxidation of (*S*)-**5** with L-DIPT showed poor stereoselectivity and under optimised conditions the resultant α,β-epoxy-alcohols **7a** and **7b** were obtained in a 3:1 ratio. Epoxidation reactions with *m*-CPBA and Ti(OiPr)_4_ gave diastereomeric ratios of between 2:1 and 3:1, thus L-DIPT showed only a modest improvement in the stereoselectivity. These diastereoisomers were not easily separated by chromatography, however the absolute and relative stereochemistry of the crystalline *threo*-isomer (*2R,3S,4S*)-**7a** was confirmed by X-ray structure analysis as shown in Figure 2.

With a strategy to access both stereoisomeric series of the allylic alcohol epoxides **A** in place, the fluorination reactions were then explored.

**Scheme 4:**
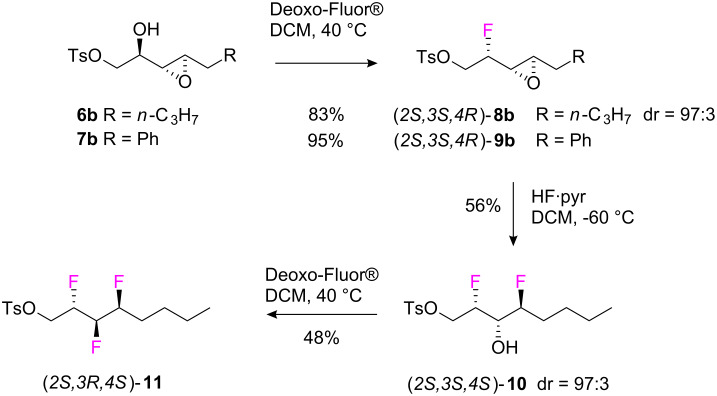
Three step sequential fluorination from α,β-epoxy alcohols to eg. the vicinyltrifluoro tosylate **11**.

The fluorination of the *anti*-isomers **6b** and **7b** was attempted using Deoxo-Fluor^®^ [17]. α,β-Epoxy-alcohol **7b** reacted smoothly with Deoxo-Fluor^®^ at 40 °C (Scheme 4) to give fluoroepoxide (*2S,3S,4R*)-**8b** in 83% yield and with a 97:3 dr. Epoxide ring opening of **8b** was then explored with HF/pyridine and this reaction proved to be both regio- and stereo-selective [18–19]. When the reaction was carried out at 0 °C the resultant difluoro alcohol **10** was obtained in 36% yield, whereas the yield improved as the temperature was lowered (47% at −35 °C and 56% at −60 °C). Epoxide ring opening was stereospecific, and the (*2S,3S,4S*)-difluoro alcohol **10** was obtained as the major diastereoisomer in a 97:3 ratio. The third fluorine atom was inserted in a smooth reaction by treatment of **10** again with Deoxo-Fluor^®^ to generate (*2S,3R,4S*)-**11**. The sequence illustrated in Scheme 4 validated the three step fluorination protocol for this diastereoisomeric series.

**Scheme 5:**
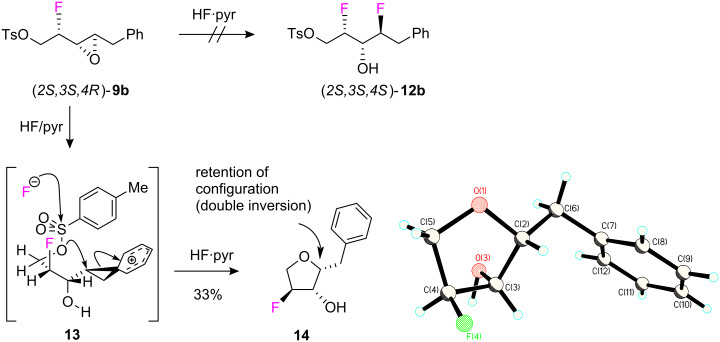
Unexpected cyclisation of **9b** to furan **14** with HF·pyridine. An X-ray structure of **14** (CCDC 750309) reveals that the cyclisation proceeds with a retention of configuration.

Reaction of α,β-epoxy alcohol **7b** with Deoxo-Fluor^®^ also proceeded smoothly generating fluoro epoxide (*2S,3S,4R*)-**9b** in 95% yield and as a single stereoisomer. However when **9b** was treated with HF/pyridine [19–20] there was no evidence that the expected difluoro alcohol **12b** had formed (Scheme 5). Instead the fluorinated tetrahydrofuran **14** was isolated as a crystalline product in 33% yield and its structure and stereochemistry were established by X-ray structure analysis (Scheme 5). This deviant reaction was surprising not only because there was no trace of the analogous cyclisation product after treatment of **8b** with HF/pyridine, but also because cyclisation had occurred with retention of configuration at C4. One explanation for this outcome is that the reaction proceeds *via* a bicyclic phenoxonium intermediate **13** generated after HF promoted epoxide ring opening. Fluoride ion triggered tosyl cleavage generates a sufficiently nucleophilic oxygen, to promote cation quenching and formation of the cyclic ether. This process would involve two configurational inversions at C4 giving overall retention.

**Scheme 6:**
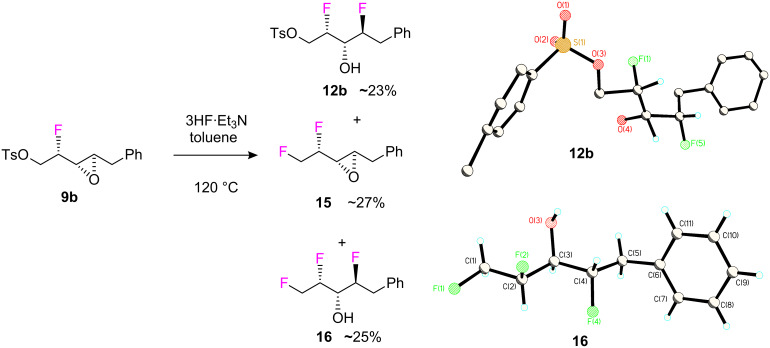
Epoxide ring opening of **9b** with 3HF·Et_3_N required forcing conditions. The structure and stereochemistry **12b** (CCDC 750308) and **16** (CCDC 750310) was established by X-ray analysis.

Epoxide ring opening of the *threo* isomer **9b** was eventually achieved, however this required much more forcing conditions using 3HF·Et_3_N in toluene at 120 °C, and generated three products **12b**, **15** and **16**, two of which arose by fluoride ion displacement of the tosyl group to generate a fluoromethyl group (Scheme 6). Interestingly there was no evidence for the formation of **14** with this less acidic reagent.

**Scheme 7:**
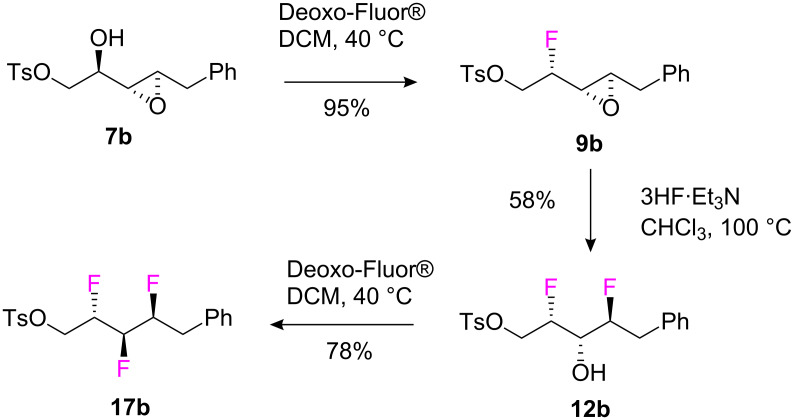
Three step sequential fluorination from α,β-epoxy alcohol **7b** to vicinal trifluoro tosylate **17b**.

Compounds **12b** and **16** were isolated in 23% and 25% yields respectively, and the stereochemistry of each was established by X-ray structure analysis. Epoxide ring opening occurred in each case in a regio- and stereo-selective manner with the expected inversion of configuration, and thus there was no evidence for the involvement of an intermediate phenoxonium ion under these conditions. The ring opening reaction of **9b** was explored under a variety of conditions and the best conversion and selectivity was obtained using chloroform at 100 °C in a sealed autoclave with a Teflon inner layer (**12b** 58%, **15** 2% and **16** 4%). For the final step of the sequence shown in Scheme 7, difluoro alcohol **12b** reacted smoothly with Deoxo-Fluor^®^ to generate trifluoroalkane **17b** which could be isolated in 78% yield.

Fluorination of epoxy alcohol **7b** by the three step protocol (44% overall yield), illustrates a second substrate of this diastereoisomeric series, and demonstrates a reasonably efficient protocol to the vicinal trifluoro motif, much improved over the original six step sequence [11–12].

Fluorination of the *threo*-α,β-epoxy alcohol **7a** proved more challenging because of a propensity to give isomeric fluorination products. Due to the difficulty in purifying the diastereoisomerically pure epoxide a deshydroxyfluorination reaction on a 1:1 mixture of **7a** : **7b** was explored. Grée and co-workers have also noticed that *erythro*-epoxy alcohols react relatively smoothly but that *threo*-epoxy alcohols are prone to rearrangement [21–22]. Thus the mixture of **7a** and **7b** was treated with Deoxo-Fluor^®^ or DAST under a variety of conditions and the outcomes are summarised in Table 1.

**Table 1:** Reaction of α,β-epoxy alcohol **7a** and **7b** under various fluorination conditions. Ratios determined by ^19^F NMR.

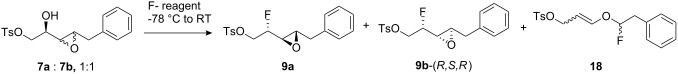			
Conditions	**9a**	**9b**	**18**

Deoxo-Fluor^®^, DCM	1	1.43	0.56
Deoxo-Fluor^®^, toluene	1	1.41	0.53
DAST, DCM	1	1.64	0.66
Deoxo-Fluor^®^, THF	1	1.29	0.44

**Scheme 8:**
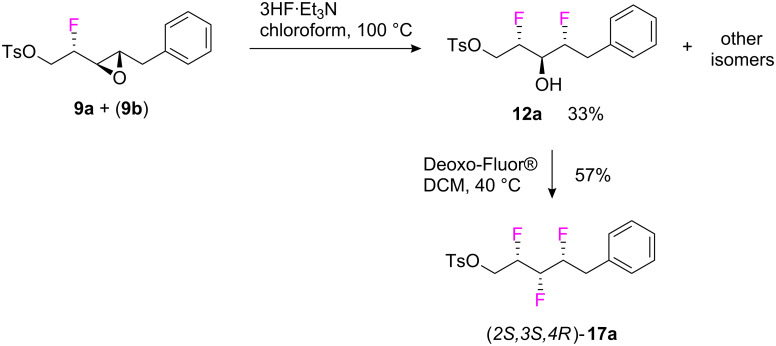
Epoxide ring opening with 3HF∙Et_3_N and synthesis of the all-*syn* vicinal trifluoro tosylate **17a**.

With this inseparable mixture of **9a** and **9b** (1:1.3 ratio) in hand, a reaction to explore the introduction of the second fluorine was carried out as illustrated in Scheme 8. Accordingly the mixture was treated with 3HF·Et_3_N in chloroform at 100 °C and this generated **12a** among other products.

Separation of (*2S,3R,4R*)-**12a** was achieved from the product mixture by chromatography (preparative TLC) in 33%. Finally, treatment of **12a** with Deoxo-Fluor^®^ gave the desired *all-syn*-trifluoro alkane (*2S,3S,4R*)-**17a** as a single enantiomer in moderate 57% yield. In general the vicinal trifluoro compounds were stable and showed no tendency to degrade over time.

## Conclusion

In summary a direct three step route has been developed from α,β-epoxy alcohols for the synthesis of the vicinal trifluoro motif where a fluorine atom is introduced in each step. Two different diastereoisomeric series (**a** and **b**) of the trifluoro motif were explored. The diastereoisomeric series **Db** (Scheme 2) could be prepared in a relatively straightforward manner, however the diastereoisomeric series **Da** (Scheme 2), where all three fluorines are *syn* with respect to an extended alkyl chain, proved to be more challenging. This is due to a greater difficulty in obtaining diastereoisomerically pure *syn*-α,β-epoxy alcohols, and also a greater propensity to side product formation during the first two fluorination reactions. Nonetheless the methods provide a direct route to this largely unexplored motif, and in the cases exemplified the synthesis delivers a product carrying a terminal tosyl ester, which should allow the vicinal trifluoro motif to be incorporated into larger molecular architectures.

## Experimental

Selected experimental data is presented. Full details are in Supporting Information.

### (2*S*,3*R*,4*S*)-2,3,4-Trifluorooctyl 4-methylbenzenesulfonate (**11**)

Deoxo-Fluor^®^ (55 μL of solution 50% in THF, 0.15 mmol) was added to a solution of **10** (25 mg, 0.07 mmol) in DCM (3 mL), and the reaction was heated at 40 °C for 1 h. The reaction was then cooled to RT and was quenched by the addition of silica gel. DCM was then removed under reduced pressure and the product was purified over silica (hexane 8/EtOAc 2) and was recovered as a colourless oil (12 mg, 48%).

[α]_D_^20^ = −2.7 (*c* = 1, CHCl_3_). ^1^H NMR (CDCl_3_, 400 MHz): δ (ppm) 7.81 (d, 2 H, *J* = 8.3 Hz, C*H* ar); 7.36 (d, 2 H, *J* = 8.3 Hz, C*H* ar); 4.86 (m, 1 H, *J* = 45.5 Hz, FC*H*); 4.74–4.37 (m, 3H, 2 × FC*H* + O_2_SOC*Ha*Hb); 4.27 (dddd, 1 H, *J* = 2.6, 4.2, 11.9, 29.6 Hz, O_2_SOCHa*Hb*); 2.46 (s, 3 H, C*H*_3_); 1.94–1.84 (m, 1 H, CHa*Hb*); 1.70–1.58 (m, 1 H, C*Ha*Hb); 1.49–1.33 (m, 4 H, 2 × C*H*_2_); 0.92 (t, 3 H, *J* = 7.4 Hz, C*H*_3_). ^13^C NMR (CDCl_3_, 100 MHz): 145.3 (*C* ar); 132.3 (*C* ar); 130.0 (*CH* ar); 128.0 (*C*H ar); 89.8 (ddd, *J* = 178.3, 18.7, 1.9 Hz, *C*F); 88.3 (ddd, *J* = 181.7, 18.6, 29.1 Hz, *C*F); 85.7 (ddd, *J* = 179.0, 30.4, 5.6 Hz, *C*F); 67.4 (d, *J* = 19.8 Hz, *C*-*C*H_2_); 29.6 (dd, *J* = 21.1, 4.6 Hz, *C*H_2_); 27.0 (d, *J* = 5.0 Hz, *C*H_2_); 22.3 (*C*H_2_); 21.6 (*C*H_3_); 13.8 (*C*H_3_). ^19^F NMR (CDCl_3_, 376 MHz): −199.82 to −200.12 (m, 1 F); −201.04 to −201.38 (m, 1 F), −214.61 to −214.89 (m, 1 F). ^19^F {^1^H} NMR (CDCl_3_, 376 MHz): −199.9 (dd, 1 F, *J* = 15.9, 3.2 Hz); −201.2 (dd, 1 F, *J* = 9.5, 3.2 Hz); −214.7 (dd, 1 F, *J* = 9.5, 15.9 Hz). ν_max_/cm^−1^ 1363, 1275, 1179, 897, 758, 749. *m/z* (ES+) = 361.01 (MNa^+^, 100%); HRMS (ES+) found 361.1047 for C_15_H_22_F_2_NaO_4_S, requires 361.1061.

### (2*S*,3*R*,4*S*)-2,3,4-Trifluoro-5-phenylpentyl 4-methylbenzenesulfonate (**17b**)

Deoxo-Fluor^®^ (50% in THF, 175 μL, 0.47 mmol) was added to a solution of **12b** (58 mg, 0.16 mmol) in DCM (3 mL) at RT. The reaction mixture was then heated at 40 °C for 1 h, and the reaction was quenched by the addition of silica gel. Solvents were removed under reduced pressure, and the product was purified over silica (hexane 5 /DCM 3 /Et_2_O 1). The title compound was recovered as a colourless oil (45 mg, 78%).

[α]_D_^20^ = −2.8 (*c* = 0.7, CDCl_3_). ^1^H NMR (CDCl_3_, 300 MHz): δ (ppm) 7.78 (d, 2 H, *J* = 8.4 Hz, C*H* ar); 7.35 (d, 2 H, *J* = 8.4 Hz, C*H* ar); 7.31–7.21 (m, 5 H, C*H* ar); 4.88 (m, 1 H, *J* = 45.9 Hz, *H*CF); 4.73–4.38 (m, 2 H, 2 × *H*CF); 4.42 (ddd, 1 H, *J* = 1.9, 12.1, 23.4 Hz, SO_3_C*Ha*Hb); 4.26 (ddd, 1 H, *J* = 4.5, 12.1, 28.9 Hz, SO_3_CHa*Hb*); 3.21 (ddd, 1 H, *J* = 7.5, 13.8, 22.2 Hz, C*Ha*HbPh); 3.01 (ddd, 1 H, *J* = 6.8, 13.8, 21.1 Hz, CHa*Hb*Ph); 2.46 (s, 3 H, C*H*_3_). ^13^C NMR (CDCl_3_, 75 MHz): 145.2 (*C* ar); 135.1 (*C* ar); 132.3 (*C* ar); 129.9 (2 *C*H ar); 129.3 (*C*H ar); 128.9 (*C*H ar); 128.0 (*C*H ar); 127.2 (*C*H ar); 90.1 (ddd, *J* = 181.8, 20.0, 2.3 Hz, *C*F); 87.0 (ddd, *J* = 183.0, 29.7, 18.1 Hz, *C*F); 86.0 (ddd, *J* = 179.5, 30.7, 5.5 Hz, *C*F); 67.4 (d, *J* = 19.7 Hz, *C*H_2_); 36.3 (dd, *J* = 22.5, 5.2 Hz, *C*H_2_); 21.7 (*C*H_3_). ^19^F NMR (CDCl_3_, 376 MHz): −198.80 to −199.23 (m, 1 F); −200.27 to −200.69 (m, 1 F), −215.48 to −215.78 (m, 1 F). ^19^F {^1^H} NMR (CDCl_3_, 376 MHz): −198.61 (dd, 1 F, *J* = 9.6, 2.8 Hz); −200.03 (dd, 1 F, *J* = 15.4, 2.8 Hz); −215.18 (dd, 1 F, *J* = 9.6, 15.4 Hz). ν_max_/cm^−1^ 1365, 1261, 1267, 1208, 1190, 1177, 1151, 749. *m/z* (ES+) = 394.95 (MNa^+^, 100%); HRMS (ES+) found 395.0906 for C_18_H_19_F_3_NaO_3_S, requires 395.0905.

### (2*S*,3*S*,4*R*)-2,3,4-Trifluoro-5-phenylpentyl 4-methylbenzenesulfonate (**17a**)

Deoxo-Fluor^®^ (50% in THF, 15 μL, 0.05 mmol) was added to a solution of **12a** (7 mg, 0.02 mmol) in DCM (1 mL) at RT. The reaction mixture was then heated at 40 °C for 1 h and the reaction was quenched by the addition of silica gel. Solvents were removed under reduced pressure and **17a** was purified by preparative TLC (hexane 7/Et_2_O 3) and recovered as a colourless oil (4 mg, 57%).

[α]_D_^20^ = +1.75 (*c* = 0.4, CDCl_3_). ^1^H NMR (CDCl_3_, 300 MHz): δ (ppm) 7.74 (d, 2 H, *J* = 8.3 Hz, *H* ar); 7.37–7.22 (m, 7 H, *H* ar); 5.06–4.43 (m, 3 H, 3 × *H*CF); 4.73–4.38 (m, 2 H, 2 × *H*CF); 4.35 (brd, 1 H, *J* = 3.8 Hz, SO_3_C*Ha*Hb); 4.27 (brd, 1 H, *J* = 3.8 Hz, SO_3_CHa*Hb*); 3.15–2.97 (m, 2 H, C*H*_2_Ph); 2.45 (s, 3 H, C*H*_3_). ^13^C NMR (CDCl_3_, 75 MHz): 130.1 (*C*H ar); 129.3 (*C*H ar); 128.9 (*C*H ar); 128.0 (*C*H ar); 127.3 (*C*H ar); 21.7 (*C*H_3_). Quaternary carbons and carbons coupled bound to fluorine were not observed, even with an extended number of scans (12000 scans). ^19^F NMR (CDCl_3_, 376 MHz): −195.84 to −196.29 (m, 1 F); −201.07 to −201.51 (m, 1 F), −212.23 to −212.63 (m, 1 F). ^19^F {^1^H} NMR (CDCl_3_, 376 MHz): −196.1 (d, 1 F, *J* = 10.8 Hz); −201.29 (d, 1 F, *J* = 13.6 Hz); −212.43 (dd, 1 F, *J* = 10.8, 13.6 Hz). ν_max_/cm^−1^ 1360, 1269, 1208, 1190, 1146, 983, 910, 740. *m/z* (ES+) = 394.96 (MNa^+^, 100%); HRMS (ES+) found 395.0903 for C_18_H_19_F_3_NaO_3_S, requires 395.0905.

## Supporting Information

File 1 Experimental methods and full characterisation and spectral data of all prepared compounds.
