# Supplementary material for: Three step synthesis of single diastereoisomers of the vicinal trifluoro motif
Source: Beilstein J Org Chem. 2009 Nov 5;5:61. doi: 10.3762/bjoc.5.61 (PMC2839549; doi:10.3762/bjoc.5.61)
Supplement: File 1 — Experimental methods and full characterisation and spectral data of all prepared compounds. [file Beilstein_J_Org_Chem-05-61-s001.doc]

**Supplementary Information**

# Synthesis of single diastereoisomers of the vicinal trifluoro motif

Vincent A. Brunet, Alexandra M. Z. Slawin and David O’Hagan*

Address: School of Chemistry and Centre for Biomolecular Sciences , University of St Andrews, North Haugh, St Andrews, Fife, KY16 9ST, UK.

Email: David O'Hagan - do1@st-andrews.ac.uk

* Corresponding author

General Methods

Nuclear magnetic resonance (NMR) spectra were obtained using Bruker Advance 300 and Bruker Advance II 400. All chemical shifts (*δ*) are reported in parts per million (ppm) and coupling constants (*J*) are given in Hertz (Hz). Melting points were determined in Pyrex capillaries using a Gallenkamp Griffin MPA350.BM2.5 melting point apparatus. Infra red (IR) spectra were recorded on Nicolet Avatar 360 FT-IR. High-mass resolution spectrometry (HRMS) was carried out using a Micromass LCT (Manchester, UK) mass spectrometer with electrospray ionization (ESI) operating in positive and negative modes. Optical rotations were measured on Perkin-Elmer model 341 polarimeter.

**(*S*)-But-3-ene-1,2-diol:** (*S*)-**2**

Butadiene monoxide (**1**) 98% (15 mL, 0.182 mol) was added to Jacobsen’s catalyst Co^III^ acetate, prepared following a literature procedure [1] (0.55 g, 0.091 mmol), and the mixture was cooled to 0 °C. Water (1.48 mL, 82 mmol) was then added dropwise and the temperature of the mixture allowed to rise to RT. After 12 h the excess of water and butadiene monoxide were distilled off under reduced pressure at 30 °C, and the title compound was recovered as a colourless oil by distillation at 60 °C (6.91g, 42%).

**[α]_D_^20^** = −45 (*c* = 2.5, iPrOH) (literature [α]_D_ −44, [1]). **^1^H-NMR** (CDCl_3_, 300 MHz): δ (ppm) 5.86 (ddd, 1 H, *J =* 5.6, 10.5, 17.3 Hz, *H*C=CHH); 5.37 (ddd, 1 H, *J =* 1.4, 17.3, 1.4 Hz, HC=C*H*H); 5.24 (ddd, 1 H, *J =* 10.6, 1.4, 1.4 Hz, HC=CH*H*); 4.29–4.23 (m, 1H, *H*COH); 3.67 (dd, 1 H, *J =* 11.2, 3.5 Hz, HOC*H*H); 3.50 (dd, 1 H, *J =* 11.2, 7.2 Hz, HOCH*H*); 2.13 (s, 2 H, OH). **^13^C-NMR** (CDCl_3_, 75 MHz): δ (ppm) = 136.7 (H*C*=CH_2_), 116.7 (HC=*C*H_2_), 73.3 (H*C*OH), 66.2 (H_2_*C*OH).

**(*S*)-2-Hydroxybut-3-enyl 4-methylbenzenesulfonate** (*S*)-**3:**

Dibutyltin oxide 98% (0.17 g, 0.67 mmol) was added to a solution of diol (*S*)-**2** (1 g, 11.33 mmol) in MeCN (20 mL), and the mixture was heated under reflux till dissolution of the dibutyltin oxide. The solution was then cooled to RT and Et_3_N (1.8 mL, 12.94 mmol) was added, followed by TsCl (2.43 g, 12.49 mmol). After 1 h at RT, the MeCN was removed under reduced pressure and the title compound was purified over silica gel (DCM 95/Et_2_O 5) and recovered as a white solid (2.03 g, 74%).

**Mp** = 58–60 °C. **[α]_D_^20^** = −7.8 (*c* = 1, MeOH) (Sigma-Aldrich, CAS number 133095-74-6, [α]_D_ −7.7). **^1^H-NMR** (CDCl_3_, 300 MHz): δ (ppm) 7.8 (d, 2 H, *J =* 8.3 Hz, *H* ar) ; 7.36 (d, 2 H, *J =* 8.3 Hz, *H* ar) ; 5.75 (ddd, 1 H, *J =* 5.4, 10.5, 17.2 Hz, *H*C=CH_2_); 5.37 (ddd, 1 H, *J =* 1.4, 17.2, 1.4 Hz, HC=C*H*H); 5.25 (ddd, 1 H, *J =* 10.5, 1.4, 1.4 Hz, HC=CH*H*) ; 4.43–4.37 (m, 1 H, *H*COH ); 4.07 (dd, 1 H, *J =* 10.2, 3.3 Hz, TsOC*Ha*Hb) ; 3.91 (dd, 1 H, *J =* 10.2, 7.4 Hz, TsOCHa*Hb*) ; 2.45 (s, 3 H, C*H_3_*); 2.15 (brs, 1 H, OH). **^13^C-NMR** (CDCl_3_, 75 MHz): δ (ppm) = 145.1 (*C* ar); 134.6 (H*C*=CH_2_); 132.6 (*C* ar); 130.0 (*C*H ar); 128.0 (*C*H ar); 118.1 (HC=*C*H_2_); 73.0 (TsO-*C*H_2_); 70.4 (HO-*C*H); 21.7 (CH_3_).

**(*S,E*)-2-Hydroxyoct-3-enyl 4-methylbenzenesulfonate:** (*S*)-**4**

Grubbs’ 2^nd^ generation catalyst (105 mg, 0.124 mmol) was added to a solution of (*S*)-**3** (1.5 g, 6.2 mmol) and 1-hexene (7.75 mL, 62.2 mmol) in DCM (15 mL). The solution was heated under reflux for 9 h and then DCM was removed under reduced pressure and the product purified over silica (DCM 95/Et_2_O 5). The title compound was recovered as a brown oil (1.20 g, 81%), in a 92:8 *E:Z* ratio.

**[α]_D_^20^** = +22.1 (*c* = 1, DCM). **^1^H-NMR** (CDCl_3_, 300 MHz): δ (ppm) 7.80 (d, 2 H, *J* = 8.4 Hz, H ar); 7.35 (d, 2 H, *J* = 8.4 Hz, H ar); 5.77 (dtd, 1 H, *J* = 15.4, 6.7, 1.1 Hz, HC=C*H*CH_2_); 5.33 (ddt, 1 H, *J* = 15.4, 6.5, 1.5 Hz, *H*C=CHCH_2_); 4.33 (m, 1 H, HOC*H*); 4.02 (dd, 1 H, *J* = 10.1, 3.4 Hz, O_2_SOC*H*H); 3.88 (dd, 1 H, *J* = 10.1, 7.7 Hz, O_2_SOCH*H*); 2.45 (s, 3 H, C*H_3_*); 2.04–1.97 (m, 2 H, C*H_2_*); 1.34–1.23 (m, 4 H, 2 × C*H_2_*); 0.87 (t, 3 H, *J* = 7.1 Hz, C*H_3_*). **^13^C-NMR** (CDCl_3_, 75 MHz): 145.0 (*C* ar); 135.6 (H*C*=CH); 132.7 (*C* ar); 129.9 (*C*H ar); 128.0 (*C*H ar); 126.2 (HC=*C*H); 73.4 (O_2_SO*C*H_2_); 70.3 (HO*C*H); 31.9 (*C*H_2_); 30.9 (*C*H_2_); 22.1 (*C*H_2_); 21.6 (*C*H_3_); 13.9 (*C*H_3_). **ν_max_/cm**^−^**^1^** 2360, 1205, 1149, 758, 764, 749. ***m/z*** (ES+) = 321.02 (MNa^+^, 100%); HRMS (ES+) calc for C_15_H_22_NaO_4_S = 321.1137 found = 321.1128.

**(*S*,*E*)-2-Hydroxy-5-phenylpent-3-enyl 4-methylbenzenesulfonate:** (*S*)-**5**

Grubbs’ 2^nd^ generation catalyst (79 mg, 0.09 mmol) was added to a solution of (*S*)-**3**

(2 g, 8.26 mmol) and allyl benzene (5.47 mL, 41.30 mmol) in DCM (20 mL). The solution was heated under reflux for 2 days and then DCM was removed under reduced pressure and the product purified over silica (DCM 95/Et_2_O 5). The title compound was recovered as a brown oil (2.20 g, 80% yield), in a 94:6 *E*:*Z* ratio.

**[α]_D_^20^** = +16.2 (*c* = 1, DCM). **^1^H-NMR** (CDCl_3_, 300 MHz): δ (ppm) 7.79 (d, 2 H, *J* = 8.3 Hz, *H* ar); 7.34 (d, 2 H, *J =* 8.3 Hz, *H* ar); 7.32–7.12 (m, 5 H, *H* ar); 5.95 (ddt, 1 H, *J*  1.2, 6.7, 15.4 Hz, *H*C=CH); 5.41 (ddt, 1 H, *J =* 1.5, 6.4, 15.4 Hz, HC=C*H*); 4.41–4.35 (m, 1 H, HOC*H*), 4.05 (dd, 1 H, *J =* 3.4, 10.2 Hz, SO_2_OC*H*H), 3.91 (dd, 1 H, *J =* 7.5, 10.2 Hz, SO_2_OCH*H*); 3.36 (d, 2 H, *J =* 6.7 Hz, C*H*_2_); 2.45 (s, 3 H, C*H*_3_), 2.11 (s, 1 H, O*H*). **^13^C-NMR** (CDCl_3_, 75 MHz): δ (ppm) = 145.5 (*C* ar); 139 (*C* ar); 134.1 (H*C*=CH); 133.1 (*C* ar); 130.41 (HC=*C*H); 129.0 (*C*H ar); 128.9 (*C*H ar); 128.4 (*C*H ar); 128.2 (*C*H ar); 126.7 (*C*H ar); 73.7 (SO_3_-*C*H_2_); 70.4 (HO-*C*H); 39.0 (*C*H_2_); 22.1 (*C*H_3_). **ν_max_/cm**^−^**^1^** 3025, 1355, 1189, 1175, 1148, 967, 750. ***m/z*** (ES+) = 355.06 (MNa^+^, 100%); HRMS (ES+) found 355.0969 for C_18_H_20_NaO_4_S, requires 355.0980.

**(*R*)-2-[(2*R*,3*R*)-3-Butyloxiran-2-yl]-2-hydroxyethyl 4-methylbenzenesulfonate: 6b**

(−)-D-diisopropyl tartrate 98% (0.16 mL, 0.738 mmol) was added to a solution of titanium tetraisopropoxide 99.99% (0.2 mL, 0.682 mmol) in DCM (7 mL) with molecular sieves 4Å at −30 °C, and the solution was stirred for 30 min at −30 °C. A solution of (*S*)-**4** (0.195 g, 0.625 mmol) in DCM (3 mL) was then added dropwise, followed by CHP 88% (0.22 mL, 1.25 mmol). The reaction mixture was stirred for 40 h at −30 °C and was then quenched by addition of a 10% solution of Na_2_S_2_O_3_. Organics were extracted into DCM (3 × 20 mL) and washed with HCl 1N (5 mL), and water (10 mL). The organic layer was dried over MgSO_4_, and solvent was removed under reduced pressure. The product was purified twice over silica gel (hexane 7/EtOAc 3 and DCM 9/Et_2_O 1) and was recovered as a colourless oil (162 mg, 80%).

**[α]_D_^20^** = +16.3 ° (c = 1, DCM). **^1^H-NMR** (CDCl_3_, 300 MHz): δ (ppm) 7.80 (d, 2 H, *J* = 8.4 Hz, H ar); 7.36 (d, 2 H, *J* = 8.4 Hz, H ar); 4.16 (dd, 1 H, *J* = 10.5, 3.9 Hz, O_2_SOC*H*H); 4.09 (dd, 1 H, *J* = 10.5, 5.5 Hz, O_2_SOCH*H*); 3.87 (m, 1 H, HOC*H*); 2.94 (td, 1 H, *J* = 5.6, 2.2 Hz, OC*H*CH_2_); 2.79 (dd, 1 H, *J* = 4.5, 2.2 Hz, OC*H*); 2.45 (s, 3 H, C*H_3_*); 2.31 (d, 1 H, *J* = 3.74 Hz, O*H*); 1.57–1.50 (m, 2 H, C*H*_2_); 1.42–1.30 (m, 4 H, 2 × CH_2_); 0.89 (t, 3 H, *J* = 7.1 Hz, C*H_3_*). **^13^C-NMR** (CDCl_3_, 75 MHz): 145.6 (*C* ar); 132.8 (*C* ar); 130.4 (*C*H ar); 128.4 (*C*H ar); 71.2 (O_2_SO*C*H_2_); 68.5 (HO*C*H); 57.6 (*C*-O); 56.7 (*C*-O); 31.4 (*C*H_2_); 28.3 (*C*H_2_); 22.8 (*C*H_2_); 22.1 (*C*H_3_); 14.3 (*C*H_3_). **ν_max_/cm**^−^**^1^** 2925, 2360, 1354, 1123, 972, 902, 749. ***m/z*** (CI+) = 315.13 (MH^+^, 10%), 297.12 (M −H_2_O, 10%), HRMS (CI) calc for C_15_H_23_O_5_S = 315.1266 found = 315.1274.

**(*R*)-2-[(2*R*,3*R*)-3-Benzyloxiran-2-yl]-2-hydroxyethyl 4-methylbenzenesulfonate 7b:**

Titanium tetraisopropoxide (0.53 mL, 1.81 mmol) was added to (−)-D-diisopropyl tartrate (0.39 mL, 1.81 mmol) in DCM (15 mL) at −30 °C with molecular sieves 4 Å. After 45 min, a precooled (−30 °C) solution of allylic alcohol (*S*)-**5** (0.5 g, 1.51 mmol) in DCM (5 mL) was slowly canulated into the reaction. CHP 88% (0.5 mL, 3.01 mmol) was then added dropwise and the reaction was stirred at −30 °C for 42 h. A solution of sodium bisulfite (10 mL) was then added and the solution warmed to RT. The organics were extracted into DCM (3 × 10 mL), washed with water (10 mL) and dried over MgSO_4_. After solvent removal the product was purified over silica (DCM 92/ Et_2_O 8) and was recovered as colourless oil (0.468 g, 90%), that slowly crystallised on standing.

**Mp** = 64–66 °C. **[α]_D_^20^** = + 24.7 (*c* = 1.05, DCM). **^1^H-NMR** (CDCl_3_, 300 MHz): δ (ppm) 7.78 (d, 2 H, *J* = 8.3 Hz, H ar); 7.51 (d, 2 H, *J* = 8.3 Hz, H ar); 7.37–7.21 (m, 5 H, *H* ar); 4.14 (dd, 1 H, *J* = 3.9, 10.6 Hz, SO_3_C*Ha*Hb); 4.08 (dd, 1 H, *J* = 5.5, 10.6 Hz, SO_3_CHa*Hb*); 3.90–3.85 (m, 1 H, HOC*H*); 3.19 (ddd, 1 H, *J* = 2.2, 4.6, 6.0 Hz, *H*C-O); 2.93 (dd, 1 H, *J* = 4.6, 14.7 Hz, PhCHa*Hb*); 2.87 (dd, 1 H, *J* = 2.2, 4.4 Hz, *H*C-O); 2.84 (dd, 1 H, *J* = 6.0, 14.7 Hz, PhC*H*H); 2.45 (s, 3 H, C*H*_3_); 2.19 (1 H, d, *J* = 3.7 Hz, O*H*). **^13^C-NMR** (CDCl_3_, 75 MHz): 145.2 (*C* ar); 136.6 (*C* ar); 132.5 (*C* ar); 130.0 (*C*H ar); 129.0 (*C*H ar); 128.6 (*C*H ar); 128.0 (*C*H ar); 126.8 (*C*H ar); 70.6 (SO_3_-*C*H_2_); 68.1 (HO-*C*H); 56.9 (H*C*O); 56.2 (H*C*O); 37.6 (*C*H_2_); 21.7 (*C*H_3_). **ν_max_/cm**^−^**^1^** 2922, 1597, 1495, 1357, 1175, 977, 749. ***m/z*** (ES+) = 370.97 (MNa^+^, 100%); HRMS (ES+) found 371.0939 for C_18_H_20_NaO_5_S, requires 371.0929.

**(*S*)-2-[(2*S*,3*R*)-3-Butyloxiran-2-yl]-2-fluoroethyl 4-methylbenzenesulfonate: 8b**

Deoxo-Fluor® (1.8 mL of solution 50% in THF, 4.48 mmol) was added to a solution of epoxy-alcohol **6b** (0.33 g, 1.05 mmol) in DCM (7 mL) and the solution was heated at 40 °C for 1 h. The reaction was then cooled to RT and was quenched by the addition of silica. DCM was removed under reduced pressure and the product was purified over silica gel (hexane 7/EtOAc 3) and was recovered as a colourless oil (0.28 g, 83%).

**[α]_D_^20^ =** −2.5 (*c* = 0.9, CHCl_3_). **^1^H-NMR** (CDCl_3_, 400 MHz): δ (ppm) 7.80 (d, 2 H, *J* = 8.4 Hz, C*H* ar); 7.36 (d, 2 H, *J* = 8.4 Hz, C*H* ar); 4.48 (dddd, 1 H, *J* = 47.7, 6.16, 4.86, 3.99 Hz, FC*H*); 4.16 (ddd, 1H, *J* = 21.38, 11.35, 3.99 Hz, O_2_SOCH*H*); 4.11 (ddd, 1 H, *J* = 17.98, 11.35, 6.16 Hz, O_2_SOC*H*H); 2.86 (ddd, 1 H, *J* = 5.62, 2.19, 0.99 Hz, OC*H*); 2.84 (ddd, 1 H, *J* = 14.41, 4.86, 2.19 Hz, OC*H*); 2.46 (s, 3 H, C*H*_3_); 1.58–1.52 (m, 2 H, C*H*_2_); 1.44–1.30 (m, 4 H, 2 × C*H*_2_); 0.90 (t, 3 H, *J* = 7.1 Hz, C*H*_3_). **^13^C-NMR** (CDCl_3_, 100 MHz): 145.4 (*C* ar); 132.3 (*C* ar); 130.0 (*C*-H ar); 128.0 (*C*-H ar); 89.1 (d, *J* = 180.3 Hz, *C*-F); 68.1 (d, *J* = 27.2, H_2_*C*-CF); 55.8 (d, *J* = 22.7 Hz, O*C*-CF); 55.2 (d, *J* = 7.9 Hz, O*C*-C-CF); 31.0 (*C*H_2_); 27.8 (*C*H_2_); 22.4 (*C*H_2_); 21.7 (*C*H_3_); 13.9 (*C*H_3_). **^19^F-NMR** (CDCl_3_, 376 MHz): −196.1 (ddddd, 1 F, *J* = 47.70, 21.39, 17.98, 14.41, 0.99 Hz). **ν_max_/cm**^−^**^1^** 2957, 2931, 2862, 1598, 1456, 1363, 1190, 1177, 944, 815, 554. ***m/z*** (ES+) = 339.05 (MNa^+^, 100%); HRMS (ES+) calc for C_15_H_21_FNaO_4_S = 339.1042 found = 339.1034.

**(2*S*,3*S*,4*S*)-2,4-Difluoro-3-hydroxyoctyl 4-methylbenzenesulfonate : 10**

HF.pyridine 70/30 (0.75 mL) was added to a solution of **8b** (0.13 g, 0.42 mmol) in DCM (5 mL) at -60 °C. After 20 h at -60 °C, HF.pyridine (0.25 mL) was added until complete conversion of **8b** (by tlc) after 8 h. The reaction was then quenched by pouring into a solution of 5% NaHCO_3_ (5 mL), and the organics were extracted into DCM (3 × 5 mL). The organic layer was washed with 5% NaHCO_3_ (5 mL) and then water (5 mL), and was dried over MgSO_4_. Solvents were removed under reduced pressure and **10** was purified over silica (hexane 8/EtOAc 2) and recovered as a colourless oil (80 mg, 56%).

**[α]_D_^20^** = −13.3 (*c* = 0.9, DCM). **^1^H-NMR** (CDCl_3_, 400 MHz): δ (ppm) 7.80 (d, 2 H, *J* = 8.4 Hz, C*H* ar); 7.30 (d, 2 H, *J =* 8.4 Hz, C*H* ar); 4.88 (m, 1 H, *J* = 47.1 Hz, FC*H*); 4.45 (ddt, 1 H, *J* = 48.0, 2.7, 8.4 Hz, FC*H*); 4.31–4.25 (m, 2 H, O_2_SOC*H_2_*); 3.68–3.56 (m, 1 H, HOC*H*); 2.45 (s, 3 H, C*H*_3_); 2.15 (dd, 1 H, *J* = 1.1, 8.7 Hz, O*H*); 1.88–1.31 (m, 6 H, 3xC*H*_2_); 0.91 (t, 3 H, *J* = 7.4 Hz, C*H*_3_). **^13^C-NMR** (CDCl_3_, 100 MHz): 145.4 (*C* ar); 132.3 (*C* ar); 130 (*C*H ar); 128.0 (*CH* ar); 91.6 (dd, *J* = 172.8, 3.2 Hz, *C*F); 88.6 (dd, *J* = 177.9, 3.4 Hz, *C*F); 70.9 (dd, *J* = 25.6, 18.3 Hz, *C*OH); 68.0 (d, *J* = 27.2 Hz, *C*-COH); 31.1 (d, *J* = 20.3 Hz, FC-*C*H_2_); 26.8 (d, *J* = 2.3 Hz, FC-C-*C*H_2_); 22.4 (*C*H_2_); 21.7 (*C*H_3_); 13.9 (*C*H_3_). **^19^F-NMR** (CDCl_3_, 376 MHz): −191.37 to −191.67 (m, 1 F); −208.82 to −209.12 (m, 1 F). **ν_max_/cm**^−^**^1^** 1268, 1275, 1179, 1125, 920, 749. ***m/z*** (ES+) = 359.05 (MNa^+^, 100%); HRMS (ES+) found 359.1100 for C_15_H_22_ F_2_NaO_4_S, requires 359.1105.

**(2*S*,3*R*,4*S*)-2,3,4-Trifluorooctyl 4-methylbenzenesulfonate: 11**

Deoxo-Fluor® (55 μL of solution 50% in THF, 0.15 mmol) was added to a solution of **10** (25 mg, 0.07 mmol) in DCM (3 mL), and the reaction was heated at 40 °C for 1 h. The reaction was then cooled to RT and was quenched by the addition of silica gel. DCM was then removed under reduced pressure and the product was purified over silica (hexane 8/EtOAc 2) and was recovered as a colourless oil (12 mg, 48%).

**[α]_D_^20^** = −2.7 (*c* = 1, CHCl_3_). **^1^H-NMR** (CDCl_3_, 400 MHz): δ (ppm) 7.81 (d, 2 H, *J* = 8.3 Hz, C*H* ar); 7.36 (d, 2 H, *J* = 8.3 Hz, C*H* ar); 4.86 (m, 1 H, *J* = 45.5 Hz, FC*H*); 4.74–4.37 (m, 3H, 2xFC*H +* O_2_SOC*Ha*Hb); 4.27 (dddd, 1 H, *J* = 2.6, 4.2, 11.9, 29.6 Hz, O_2_SOCHa*Hb*); 2.46 (s, 3 H, C*H*_3_); 1.94–1.84 (m, 1 H, CHa*Hb*); 1.70–1.58 (m, 1 H, C*Ha*Hb); 1.49–1.33 (m, 4 H, 2xC*H*_2_); 0.92 (t, 3 H, *J* = 7.4 Hz, C*H*_3_). **^13^C-NMR** (CDCl_3_, 100 MHz): 145.3 (*C* ar); 132.3 (*C* ar); 130.0 (*CH* ar); 128.0 (*C*H ar); 89.8 (ddd, *J* = 178.3, 18.7, 1.9 Hz, *C*F); 88.3 (ddd, *J* = 181.7, 18.6, 29.1 Hz, *C*F); 85.7 (ddd, *J* = 179.0, 30.4, 5.6 Hz, *C*F); 67.4 (d, *J* = 19.8 Hz, *C*-*C*H_2_); 29.6 (dd, *J* = 21.1, 4.6 Hz, *C*H_2_); 27.0 (d, *J* = 5.0 Hz, *C*H_2_); 22.3 (*C*H_2_); 21.6 (*C*H_3_); 13.8 (*C*H_3_). **^19^F-NMR** (CDCl_3_, 376 MHz): −199.82 to −200.12 (m, 1 F); −201.04 to −201.38 (m, 1 F), −214.61 to −214.89 (m, 1 F). **^19^F-{^1^H}-NMR** (CDCl_3_, 376 MHz): −199.9 (dd, 1 F, *J* = 15.9, 3.2 Hz); −201.2 (dd, 1 F, *J* = 9.5, 3.2 Hz); −214.7 (dd, 1 F, *J* = 9.5, 15.9 Hz). **ν_max_/cm**^−^**^1^** 1363, 1275, 1179, 897, 758, 749. ***m/z*** (ES+) = 361.01 (MNa^+^, 100%); HRMS (ES+) found 361.1047 for C_15_H_22_ F_2_NaO_4_S, requires 361.1061.

**(*S*)-2-[(2*S*,3*R*)-3-Benzyloxiran-2-yl]-2-fluoroethyl 4-methylbenzenesulfonate: 9b**

Deoxo-Fluor® (50% in THF, 0.92 mL, 2.47 mmol) was added to a solution of epoxy-alcohol **7b** (0.29 g, 0.83 mmol) in DCM (10 mL) at RT. The reaction was heated at 40 °C for 1 h and was quenched by the addition of silica gel. Solvents were removed under reduced pressure and fluoro-epoxide **9b** was purified over silica (hexane 4/DCM 4/Et_2_O 1) and was recovered as a colourless oil (0.27 g, 95%).

**[α]_D_^20^** = +2.4 (*c* = 0.99, DCM). **^1^H-NMR** (CDCl_3_, 300 MHz): δ (ppm) 7.77 (d, 2 H, *J* = 8.4 Hz, C*H* ar); 7.37–7.20 (m, 7 H, C*H* ar); 4.58 (dddd, 1 H, *J* = 47.6, 6.2, 4.6, 3.9 Hz, *H*CF); 4.19 (ddd, 1 H, *J* = 21.9, 11.4, 3.9 Hz, SO_3_C*Ha*Hb); 4.16 (ddd, 1 H, *J* = 18.2, 11.4, 6.2 Hz, SO_3_CHa*Hb*); 3.19 (tdd, 1 H, *J* = 5.2, 2.2, 0.8 Hz, *H*CO); 2.97 (ddd, 1 H, *J* = 14.8, 4.6, 2.2, *H*CO); 2.91 (d, 2 H, *J* = 5.2 Hz, C*H*_2_Ph); 2.46 (s, 3 H, C*H*_3_). **^13^C-NMR** (CDCl_3_, 75 MHz): δ (ppm) 145.4 (*C* ar); 136.2 (*C* ar); 132.3 (*C* ar); 130.1 (*C*H ar); 129.0 (*C*H ar); 128.7 (*C*H ar); 128.0 (*C*H ar); 127.0 (*C*H ar); 88.9 (d, *J* = 181.2 Hz, *C*F); 68.2 (d, *J* = 26.6 Hz, *C*H_2_CF); 55.5 (d, *J* = 22.9 Hz, *C*HCF); 55.2 (d, *J* = 8.0 Hz, FC-CH-*C*H); 37.4 (*C*H_2_); 21.7 (*C*H_3_). **^19^F-NMR** (CDCl_3_, 282MHz): −196.61 (ddddd, 1 F, *J* = 47.6, 21.9, 18.2, 14.8, 0.8 Hz). **ν_max_/cm**^−^**^1^** 1362, 1276, 1268, 1177, 993, 749. ***m/z*** (ES+) = 373.06 (MNa^+^, 100%); HRMS (ES+) found 373.0883 for C_18_H_19_FNaO_4_S, requires 373.0886.

**(2*S*,3*S*,4*S*)-2,4-Difluoro-3-hydroxy-5-phenylpentyl 4-methylbenzenesulfonate: 12b**

3HF.Et_3_N (1 mL, 6.13 mmol) was added to a solution of fluoro epoxide **9b** (0.14 g, 0.40 mmol) in chloroform (5 mL) the reaction placed in a Teflon lined sealed bomb. The reaction was stirred at 100 °C for 3 days, and was then quenched by the addition of aqueous NaHCO_3_ 5% (5 mL). Organics were extracted into DCM (3 × 5 mL) and were washed with NaHCO_3_ 5% (5 mL) and then water (5 mL). The organic layers were dried over MgSO_4_ and solvents were removed under reduced pressure. The product was purified over silica gel (hexane 5/DCM 3/Et_2_O 2) and recovered as a white solid (86 mg, 58%).

**Mp** = 66–68 °C. **[α]_D_^20^ =** −24.2 (*c* = 0.9, DCM). **^1^H-NMR** (CDCl_3_, 300 MHz): δ (ppm) 7.79 (d, 2 H, *J* = 8.3 Hz, C*H* ar); 7.35 (d, 2 H, *J*= 8.3 Hz, C*H* ar); 7.32–7.24 (m, 5 H, C*H* ar); 4.89 (m, 1 H, *J* = 47.2 Hz, *H*CF); 4.69 (ddt, 1 H, *J* = 3.1, 8.0, 47.1 Hz, *H*CF); 4.31–4.21 (m, 2 H, SO_3_C*HaHb*); 3.68–3.58 (m, 1 H, *H*COH); 3.20 (ddd, 1 H, *J* = 3.0, 14.8, 31.7 Hz, C*Ha*HbPh); 2.97 (ddd, 1 H, *J* = 7.2, 14.8, 26.1 Hz, CHa*Hb*Ph); 2.45 (s, 3 H, C*H*_3_), 2.24 (m, 1 H, O*H*). **^13^C-NMR** (CDCl_3_, 75 MHz): 145.4 (*C* ar); 136.0 (*C* ar); 132.3 (*C* ar); 130.0 (2 *C*H ar); 129.7 (*C*H ar); 128.6 (*C*H ar); 128.0 (*C*H ar); 126.9 (*C*H ar); 91.2 (dd, *J* = 176.0, 3.7 Hz, H*C*-CF); 88.5 (dd, *J* = 177.9, 3.0 Hz, H*C*-CF); 70.1 (dd, *J* = 25.7, 18.3 Hz, HO*C*H); 67.9 (d, *J* = 27.1 Hz, *C*H_2_-CF); 37.6 (d, *J* = 20.0 Hz, *C*H_2_-CF); 21.7 (*C*H_3_). **^19^F-NMR** (CDCl_3_, 282MHz): −189.82 to −190.20 (m, HC*F*); −209.30 to −209.71 (m, HC*F*). **^19^F {^1^H}-NMR** (CDCl_3_, 282MHz): −189.6 (d, *J* = 3.5 Hz, HC*F*); −209.0 (d, *J* = 3.5 Hz, HC*F*). **ν_max_/cm**^−^**^1^** 1267, 1261, 1209, 1151, 758, 749. ***m/z*** (ES+) = 393.01 (MNa^+^, 100%); HRMS (ES+) found 393.0942 for C_18_H_20_ F_2_NaO_4_S, requires 393.0948.

**(2*R*,3*S*,4*S*)-2-Benzyl-4-fluorotetrahydrofuran-3-ol: 14**

HF.pyridine 70/30 (0.75 mL) was added to a solution of **9b** (0.115 g, 0.33 mmol) in DCM (5 mL) at 0 °C. After 6 h, the reaction was quenched by pouring into a solution of 5% NaHCO_3_ (5 mL), and organic product was extracted into DCM (3 × 5 mL). The organic layer was washed with solution of 5% NaHCO_3_ (5 mL) and then water (5 mL), and was dried over MgSO_4_. Solvents were removed under reduced pressure and the product was purified over silica gel (hexane 6/DCM 2/Et_2_O 2) and recovered as a white solid (23 mg, 33%).

**Mp** = 86–88 °C. **[α]_D_^20^** = −12.9 (*c* = 1, CHCl_3_). **^1^H-NMR** (CDCl_3_, 400 MHz): δ (ppm) 7.34–7.24 (m, 5 H, *H* ar), 5.01 (dd, 1 H, *J* = 3.7, 52.2 Hz, *H*CF); 4.27 (ddd, 1 H, *J* = 4.0, 11.4, 38.5 Hz, *H*COH); 4.24–4.12 (m, 2 H, OC*H_2_*); 3.91 (dd, 1 H, *J* = 11.5, 31.6 Hz, *H*CO); 3.07–2.95 (m, 2 H, C*H_2_*Ph); 1.84 (d, 1 H, *J* = 6.3 Hz, O*H*). **^13^C-NMR** (CDCl_3_, 100 MHz): 137.8 (*C* ar); 129.1 (*C*H ar); 128.6 (*C*H ar); 126.6 (*C*H ar); 96.8 (d, *J* = 181.6 Hz, *C*-F); 81.6 (O-*C*H-CH_2_Ph); 74.5 (d, *J* = 25.4 Hz, HO-*C*); 71.4 (d, *J* = 23.6 Hz, *C*H_2_O); 34.3 (*C*H_2_Ph). **^19^F-NMR** (CDCl_3_, 376MHz): −180.3 to −184.9 (m, 1 F). **ν_max_/cm**^−^**^1^** 3329, 1276, 1180,1055, 764, 750. ***m/z*** (ES+) = 219.01 (MNa^+^, 100%); HRMS (ES+) found 219.0794 for C_11_H_13_FNaO_2_, requires 219.0797.

**(2*R*,3*S*,4*S*)-1,2,4-Trifluoro-5-phenylpentan-3-ol: 16**

3HF.Et_3_N (1 mL, 6.13 mmol) was added to a solution of fluoro epoxide **9b** (0.05 g, 0.14 mmol) in toluene (1 mL) in a Teflon lined sealed bomb. The reaction mixture was stirred at 120 °C for 18 h and was then quenched by the addition of aqueous NaHCO_3_ 5% ( 2 mL). The organic product was extracted into DCM (3 × 5 mL), and was washed with NaHCO_3_ 5% (5 mL) and water (5mL). The organic layer was dried over MgSO_4_ and solvents were removed under reduced pressure. The title compound was purified over silica gel (hexane 5/DCM 3/Et_2_O 1) and recovered as a white solid (7 mg, 25%).

Difluoroalcohol **12b** was also recovered (11 mg) from this reaction.

**^1^H-NMR** (CDCl_3_, 300 MHz): δ (ppm) 7.35–7.26 (m, 5 H, *H* ar); 4.93 (ddm, 1 H, *J* = 18.4, 47.7 Hz, FCH_2_-C*H*F); 4.85–4.51 (m, 3 H, FC*H_2_* + HOC-C*H*F); 3.71 (m, 1 H, *J* = 23.8 Hz, *H*C-OH); 3.24 (ddd, 1 H, *J* = 3.2, 15.0, 31.3 Hz, C*Ha*HbPh); 3.01 (ddd, 1 H, *J* = 7.6, 15.0, 26.8 Hz, CHa*Hb*Ph); 2.10 (dt, 1 H, *J* = 8.3, 1.8 Hz, O*H*). **^13^C-NMR** (CDCl_3_, 75 MHz): 136.0 (*C* ar); 129.7 (*C*H ar); 128.6 (*C*H ar); 126.9 (*C*H ar); 91.3 (dd, *J* = 2.9, 175.8 Hz, *C*-F); 89.6 (ddd, *J* = 3.1, 20.2, 175.9 Hz, *C*-F); 82.4 (ddd, *J* = 2.1, 24.8, 171.6 Hz, *C*H_2_F); 70.2 (ddd, *J* = 6.1, 18.8, 26.1 Hz, *C*OH); 37.5 (d, *J* = 20.4 Hz, *C*H_2_Ph). **^19^F-NMR** (CDCl_3_, 282MHz): −189.7 to −190.1 (m, HC*F*); −210.9 to −211.4 (m, HC*F*); −232.7 to −233.2 (m, CH_2_*F*). **^19^F{^1^H}-NMR** (CDCl_3_, 282MHz): −189.9 (d, *J =* 3.0 Hz, HC*F*); −211.1 (dd, *J* = 3.0, 14.8 Hz, HC*F*); −232.9 (d, *J* = 14.8 Hz, CH_2_*F*). ***m/z*** (ES+) = 241.03 (MNa^+^, 100%); HRMS (ES+) found 241.0813 for C_11_H_13_ F_3_NaO, requires 241.0816.

**(2*S*,3*R*,4*S*)-2,3,4-Trifluoro-5-phenylpentyl 4-methylbenzenesulfonate: 17b**

Deoxo-Fluor® (50% in THF, 175 μL, 0.47 mmol) was added to a solution of **12b** (58 mg, 0.16 mmol) in DCM (3 mL) at RT. The reaction mixture was then heated at 40 °C for 1 h, and the reaction was quenched by the addition of silica gel. Solvents were removed under reduced pressure, and the product was purified over silica (hexane 5/DCM 3/Et_2_O 1). The title compound was recovered as a colourless oil (45 mg, 78 %).

**[α]_D_^20^** = −2.8 (*c* = 0.7, CDCl_3_). **^1^H-NMR** (CDCl_3_, 300 MHz): δ (ppm) 7.78 (d, 2 H, *J* = 8.4 Hz, C*H* ar); 7.35 (d, 2 H, *J* = 8.4 Hz, C*H* ar); 7.31–7.21 (m, 5 H, C*H* ar); 4.88 (m, 1 H, *J* = 45.9 Hz, *H*CF); 4.73–4.38 (m, 2 H, 2 × *H*CF); 4.42 (ddd, 1 H, *J =* 1.9, 12.1, 23.4 Hz, SO_3_C*Ha*Hb); 4.26 (ddd, 1 H, *J =* 4.5, 12.1, 28.9 Hz, SO_3_CHa*Hb*); 3.21 (ddd, 1 H, *J* = 7.5, 13.8, 22.2 Hz, C*Ha*HbPh); 3.01 (ddd, 1 H, *J* = 6.8, 13.8, 21.1 Hz, CHa*Hb*Ph); 2.46 (s, 3 H, C*H*_3_). **^13^C-NMR** (CDCl_3_, 75 MHz): 145.2 (*C* ar); 135.1 (*C* ar); 132.3 (*C* ar); 129.9 (2 *C*H ar); 129.3 (*C*H ar); 128.9 (*C*H ar); 128.0 (*C*H ar); 127.2 (*C*H ar); 90.1 (ddd, *J* = 181.8, 20.0, 2.3 Hz, *C*F); 87.0 (ddd, *J* = 183.0, 29.7, 18.1 Hz, *C*F); 86.0 (ddd, *J* = 179.5, 30.7, 5.5 Hz, *C*F); 67.4 (d, *J*= 19.7 Hz, *C*H_2_); 36.3 (dd, *J*= 22.5, 5.2 Hz, *C*H_2_); 21.7 (*C*H_3_). **^19^F-NMR** (CDCl_3_, 376 MHz): −198.80 to −199.23 (m, 1 F); −200.27 to −200.69 (m, 1 F), −215.48 to −215.78 (m, 1 F). **^19^F{^1^H} NMR** (CDCl_3_, 376 MHz): −198.61 (dd, 1 F, *J* = 9.6, 2.8 Hz); −200.03 (dd, 1 F, *J*= 15.4, 2.8 Hz); -215.18 (dd, 1 F, *J* = 9.6, 15.4 Hz). **ν_max_/cm**^−^**^1^** 1365, 1261, 1267, 1208, 1190, 1177, 1151, 749. ***m/z*** (ES+) = 394.95 (MNa^+^, 100%); HRMS (ES+) found 395.0906 for C_18_H_19_ F_3_NaO_3_S, requires 395.0905.

**(*R*)-2-[(2*S*,3*S*)-3-Benzyloxiran-2-yl]-2-hydroxyethyl 4-methylbenzenesulfonate: 7a**

Epoxy-alcohol **7a** was obtained after several consecutive recrystallisations of a ~ 1:1 mixtureof **7a** : **7b**. Epoxide **7a** was unstable on storage and was used immediately after preparation.

**^1^H-NMR** (CDCl_3_, 300 MHz): δ (ppm) 7.8 (d, 2 H, *J =* 8.1 Hz, H ar); 7.35 (d, 2 H, *J* = 8.1 Hz, H ar); 7.31–7.20 (m, 5 H, H ar); 4.07 (brs, 1 H, SO_3_C*Ha*Hb); 4.05 (brs, 1 H, SO_3_CHa*Hb*); 3.93–3.86 (m, 1 H, HOC*H*); 3.20 (ddd, 1 H, *J* = 2.2, 5.6, 5.4 Hz, *H*C-O); 2.93–2.87 (m, 3 H, PhC*H_2_* + *H*C-O); 2.45 (s, 3 H, C*H*_3_); 2.19 (1 H, d, *J* = 7.2 Hz, O*H*). ***m/z*** (ES+) = 371.05 (MNa^+^, 100%); HRMS (ES+) found 371.0939 for C_18_H_20_NaO_5_S, requires 371.0929.

**(2*S*,3*R*,4*R*)-2,4-Difluoro-3-hydroxy-5-phenylpentyl 4-methylbenzenesulfonate: 12a**

Deoxo-Fluor® (50% in THF, 0.24 mL, 0.6 mmol) was added to a solution of epoxy-alcohol **7a** and **7b** in a ~ 1:1 ratio (148 mg, 0.42 mmol) in DCM (5 mL) at RT. The reaction mixture was heated at 40 °C for 1 h, and then the reaction was quenched by the addition of silica gel. Solvents were removed under reduced pressure and fluoro-epoxides **9a** and **9b** and **18** were purified over silica gel (hexane 6/DCM 3/Et_2_O 1) and the product **9a**:**9b** recovered as a colourless oil (70 mg, 47%).

**^19^F{^1^H} NMR** (CDCl_3_, 282MHz) [-195.9] and [-197.0] in a **9a**:**9b** 1:1.3 ratio.

3HF.Et_3_N (0.5 mL, 3 mmol) was added to a solution of the fluoro epoxides **9a**:**9b** (34 mg, 0.10 mmol) in chloroform (3 mL) in a Teflon lined sealed bomb. The reaction mixture was stirred at 100 °C for 3 days and was then quenched by the addition of aqueous NaHCO_3_ 5% (5 mL). The organics were extracted into DCM (3 × 5 mL), and were washed with NaHCO_3_ 5% (5 mL) and then water (5 mL). The organic layers were dried over MgSO_4_ and the solvent was removed under reduced pressure. Chromatography allowed purification of **12a** (7 mg, 33%) as a single diastereoisomer.

**[α]_D_^20^** = +1.4 (*c* = 1, DCM). **^1^H-NMR** (CDCl_3_, 300 MHz): δ (ppm) 7.80 (d, 2 H, *J* = 8.2 Hz, C*H* ar); 7.36 (d, 2 H, *J* = 8.2 Hz, C*H* ar); 7.32–7.22 (m, 5 H, *H* ar); 4.77 (m, 1 H, *J* = 46.1 Hz, *H*CF); 4.73 (m, 1 H, *J* = 47.1 Hz, *H*CF); 4.41–4.39 (m, 1 H, SO_3_C*Ha*Hb); 4.33–4.31 (m, 1 H, SO_3_CHa*Hb*); 4.10–3.99 (m, 1 H, *H*COH); 2.97 (m, 2 H, C*H_2_*Ph); 2.45 (s, 3 H, C*H*_3_). **^13^C-NMR** (CDCl_3_, 75 MHz): 145.3 (*C* ar); 136.2 (*C* ar); 132.5 (*C* ar); 130.0 (*C*H ar); 129.4 (*C*H ar); 128.6 (*C*H ar); 128.0 (*C*H ar); 126.9 (*C*H ar); 93.4 (dd, *J*= 174.0, 4.2 Hz, *C*F); 89.6 (dd, *J* = 178.2, 5.2 Hz, *C*F); 70.1 (t, *J* = 23.6 Hz, HO*C*H); 68.1 (dd, *J* = 21.6, 3.0 Hz, *C*H_2_); 36.5 (d, *J* = 21.0 Hz, *C*H_2_); 29.7 (*C*H_3_). **^19^F-NMR** (CDCl_3_, 282MHz): −192.54 to −192.94 (m, HC*F*); −198.50 to −198.88] (m, HC*F*). **^19^F{^1^H}-NMR** (CDCl_3_, 282 MHz): −192.73 (s, HC*F*); −198.69 (s, HC*F*). **ν_max_/cm**^−^**^1^** 2921, 1360,1190, 1176, 1146, 983, 913, 743. ***m/z*** (ES+) = 392.95 (MNa^+^, 100%); HRMS (ES+) found 393.0946 for C_18_H_20_ F_2_NaO_4_S, requires 393.0948.

**(2*S*,3*S*,4*R*)-2,3,4-Trifluoro-5-phenylpentyl 4-methylbenzenesulfonate : 17a**

Deoxo-Fluor® (50% in THF, 15 μL, 0.05 mmol) was added to a solution of **12a** (7 mg, 0.02 mmol) in DCM (1 mL) at RT. The reaction mixture was then heated at 40 °C for 1 h and the reaction was quenched by the addition of silica gel. Solvents were removed under reduced pressure and **17a** was purified by preparative TLC (hexane 7/Et_2_O 3) and recovered as a colourless oil (4 mg, 57%).

**[α]_D_^20^** = +1.75 ° (c = 0.4, CDCl_3_). **^1^H-NMR** (CDCl_3_, 300 MHz): δ (ppm) 7.74 (d, 2 H, *J* = 8.3 Hz, *H* ar); 7.37–7.22 (m, 7 H, *H* ar); 5.06–4.43 (m, 3 H, 3 × *H*CF); 4.73–4.38 (m, 2 H, 2 × *H*CF); 4.35 (brd, 1 H, *J =* 3.8 Hz, SO_3_C*Ha*Hb); 4.27 (brd, 1 H, *J =* 3.8 Hz, SO_3_CHa*Hb*); 3.15–2.97 (m, 2 H, C*H_2_*Ph); 2.45 (s, 3 H, C*H*_3_). **^13^C-NMR** (CDCl_3_, 75 MHz): 130.1 (*C*H ar); 129.3 (*C*H ar); 128.9 (*C*H ar); 128.0 (*C*H ar); 127.3 (*C*H ar); 21.7 (*C*H_3_). Quaternary carbons and carbons coupled bound to fluorine were not observed, even with an extended number of scans (12000 scans). **^19^F-NMR** (CDCl_3_, 376 MHz): −195.84 to −196.29 (m, 1 F); −201.07 to −201.51 (m, 1 F), −212.23 to −212.63 (m, 1 F). **^19^F{^1^H}-NMR** (CDCl_3_, 376 MHz): −196.1 (d, 1 F, *J* = 10.8 Hz); −201.29 (d, 1 F, *J* = 13.6 Hz); −212.43 (dd, 1 F, *J* = 10.8, 13.6 Hz). **ν_max_/cm**^−^**^1^** 1360, 1269, 1208, 1190, 1146, 983, 910, 740. ***m/z*** (ES+) = 394.96 (MNa^+^, 100%); HRMS (ES+) found 395.0903 for C_18_H_19_ F_3_NaO_3_S, requires 395.0905.

## Reference

1. Schaus, S. E.; Brandes, B. D.; Larrow, J. F.; Tokunaga, M.; Hansen, K. B.; Gould, A. E.; Furrow, M. E.; Jacobsen, E. N. *J. Am. Chem. Soc.* **2002,** *124,* 1307–1315. doi:10.1021/ja016737l
